# Strain Sensor-Inserted Microchannel for Gas Viscosity Measurement

**DOI:** 10.3390/bios13010076

**Published:** 2023-01-01

**Authors:** Kota Shiba, Linbo Liu, Guangming Li

**Affiliations:** 1 Center for Functional Sensor & Actuator (CFSN), National Institute for Materials Science (NIMS), 1-1 Namiki, Tsukuba 305-0044, Ibaraki, Japan; 2 John A. Paulson School of Engineering and Applied Sciences, Harvard University, 9 Oxford Street, Cambridge, MA 02138, USA; 3 DAAN Gene Co., Ltd. of Guangzhou, 19 Xiangshan Road, Science Park, High & New Technology Development District, Guangzhou 510665, China; 4 State Key Laboratory of Rare Earth Resource Utilization, Changchun Institute of Applied Chemistry, 5625 Renmin Street, Changchun 130022, China

**Keywords:** viscosity, gas, microchannel, strain, sensor, PDMS

## Abstract

Quantifying the viscosity of a gas is of great importance in determining its properties and can even be used to identify what the gas is. While many techniques exist for measuring the viscosities of gases, it is still challenging to probe gases with a simple, robust setup that will be useful for practical applications. We introduce a facile approach to estimating gas viscosity using a strain gauge inserted in a straight microchannel with a height smaller than that of the gauge. Using a constrained geometry for the strain gauge, in which part of the gauge deforms the channel to generate initial gauge strain that can be transduced into pressure, the pressure change induced via fluid flow was measured. The change was found to linearly correlate with fluid viscosity, allowing estimation of the viscosities of gases with a simple device.

## 1. Introduction

Viscosity is one of the common properties of fluids; it is familiar to us and we have many chances to feel its presence in our daily lives. For example, water flows quickly, while honey flows relatively slowly. We can use viscosity as a rough measure to differentiate liquids without any special equipment because liquid viscosity varies over an extremely wide range, from approximately 10^−4^ to 10^8^ Pa·s [1,2]. In contrast, we are not able to differentiate gases based on their viscosities in the same manner as we are for liquids; gas is much less viscous than liquid, and viscosity values of most gases vary in a far narrower range, from 10^−6^ to 10^−5^ Pa·s, than those of liquids [1]. Moreover, gas is easy to diffuse, so typical methods for measuring liquid viscosities, such as various rotational viscometers and microchannels [3,4,5,6], are not applicable to gases. Instead, many different techniques have been developed to quantify gas viscosity, including the measurement of the differential pressure in a capillary [7,8,9,10,11,12]; the viscous drag of a falling object [13]; the damping of oscillations of a disc [14,15,16,17,18]; light scattering [19]; the change in speed of a levitated rotational disc [20]; light absorption [21]; and the shift in resonant frequency of a vibrating object, such as quartz crystal microbalance [22], a microcantilever [23,24], or a tuning fork [25]. There is, however, no standard technique, as viscometers are for liquids, conveniently used to measure gas viscosity. A simple microchannel embedded with a strain sensor is used to measure the viscosities of various fluids, although the operation is limited at a low flow rate, ranging from 5 to 10 mL/min, and the relationship between measured output and viscosity is nonlinear [26]. With the increasing demand for rapid, facile measurements using mobile devices, even outside laboratories, efforts have been made to establish techniques without bulky, complicated setup. This is still challenging after the development of a variety of analytical methods; nevertheless, this technique would enable us to measure any gases and thus discriminate them based on their viscosities, leading to various applications in safety, food, cosmetics, health, and related fields.

In this paper, we describe a very simple device that correlates pressure that drives gas flow with gas viscosity. We accomplished this via measurement of flow-induced changes in pressure as a function of flow rate. We inserted a strain gauge in a micron-sized straight channel with a height smaller than the thickness of the gauge. In this constrained geometry, the thickest part of the strain gauge deformed the channel, simultaneously generating gauge strain that could be transduced into pressure. The subsequent introduction of flow also deformed the channel and thus changed the pressure initially applied on the gauge. We demonstrated this approach by correlating changes in pressure with the viscosities of gases including carbon dioxide (CO_2_), nitrogen (N_2_), air, helium (He), and argon (Ar).

## 2. Concept

One of the established techniques for determining fluid viscosity is to use a circular channel, such as a glass capillary, to measure the pressure drop between two points through which the fluid is flowing. The fluid viscosity will be proportional to the pressure drop, and this relationship is also applicable to a fluid that flows through other channels with rectangular cross-sections after minor modifications to the analytic model [27]. We can conveniently use this relationship to estimate fluid viscosity; however, it significantly varies if we use a channel that consists of a deformable material, such as polydimethylsiloxane (PDMS) [28,29,30,31,32], which is one of the most common materials in microfluidic studies. Owing to its highly deformable feature, the pressure drop with PDMS is much less and is nonlinearly dependent on the viscosity, making it a rather inefficient way of measuring viscosity.

What we present in this paper is a different approach to fluid viscosity from those reported previously, even though we still used a PDMS microchannel. We focused on the flow-induced deformation of a channel and measured induced changes in the pressure at a certain point instead of simply measuring the deformation itself. Then, we found that there was a linear correlation between viscosity and changes in pressure. We demonstrated this approach via insertion of a strain gauge with a thickness higher than the channel height and monitoring the output-voltage changes that could be transduced into changes in pressure. This constrained geometry allowed us to obtain changes in pressure as a function of flow-induced deformation. We found via experiment that changes in pressure are proportional to fluid viscosity, and we analytically validated that relationship. Our approach of using a PDMS device is more robust against mechanical impact than is the conventional method of using a glass capillary because of the flexible features of PDMS. Furthermore, we no longer needed to monitor the pressure drop between two points, meaning that the whole setup was simpler than that of conventional techniques.

## 3. Materials and Methods

### 3.1. Device Fabrication

We used a spin coating procedure (2000 rpm for 30 s) to coat negative photoresist SU-8 3050 onto a clean silicon wafer (3-inch, single-side-polished), leading to the formation of a 60 μm-thick layer. The coated silicon wafer was baked at 95 °C for 30 min. Then, we used a printed photomask to cover the photoresist layer and expose it to UV light. The mask was removed from the wafer after the UV irradiation, and the baking process was performed again, at 95 °C for 5 min. The baked silicon wafer was washed with propylene glycol methyl ether acetate (PGMEA) for 12 min to remove the uncured resist and to obtain a master mold with microchannel structures.

**Figure 1:**
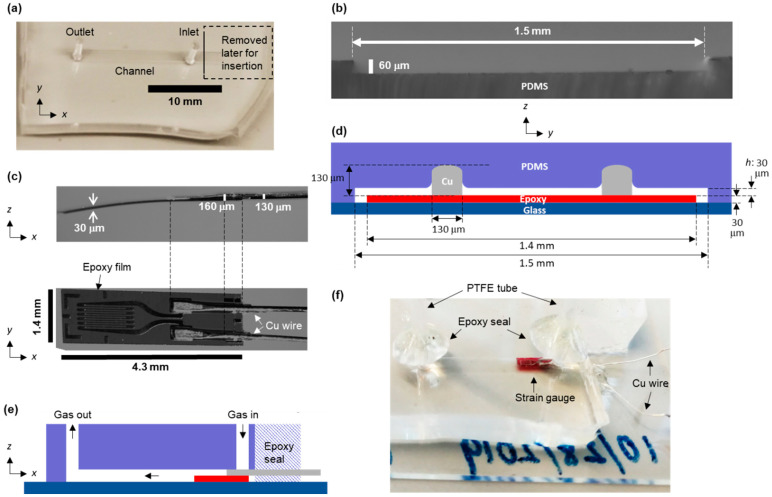
(**a**) Photo of the PDMS microchannel. (**b**) Cross-sectional image of the microchannel shown in (**a**). (**c**) Optical microscope images (side view and top view) of a strain gauge. (**d**,**e**) Cross-sectional schematic of the microchannel with an inserted strain gauge. (**f**) Photo of the PDMS device after insertion of the strain gauge and sealing it with epoxy.

Then, we adopted a micromolding procedure to replicate the microchannel from the master mold. A well-stirred polydimethylsiloxane (PDMS) mixture (weight ratio of base to curing agent set at 10:1) was poured on the master mold and degassed until no fine bubbles emerged. The PDMS mixture was cured at 65 °C for several hours. After the curing process, the PDMS was completely solidified and could be easily peeled off of the master mold. We used a biopsy punch to make through-holes for the inlet and the outlet. The PDMS block was exposed to an oxygen plasma in order to irreversibly bond it to a glass slide. We finalized the device fabrication by putting the device in an oven at 90 °C for an hour to enhance the interfacial bonding. A photo and an optical microscope image of the resulting PDMS device are shown in Figure 1a,b,f. Some of the images in Figure 1 were taken with an optical microscope (Zeiss Axio Zoom.V16 microscope; Zeiss International, Oberkochen, Germany), using an HXP 200 C metal halide illumination module (Zeiss International, Oberkochen, Germany) and a 16-bit Hamamatsu ORCA-Flash4.0 V2 Digital CMOS camera (Hamamatsu Photonics, Hamamatsu City, Japan)).

### 3.2. Gas Flow Measurement with the PDMS Device

The PDMS device was used to measure changes in pressure under the flows of various gases. For all measurements, we used a strain gauge (gauge pattern: FLKB-1-11; length: 4.3 mm; width: 1.4 mm; thickness: 30 μm; gauge factor: 2.11; gauge resistance: 120 Ω; purchased from Tokyo Measuring Instruments Laboratory Co., Ltd.). A bridge circuit for the measurements is shown in Figure S1. An optical microscope image was taken to show the exact dimensions of the strain gauge, as shown in Figure 1c. All of the output voltages from the strain gauge were measured with an NI 9237 simultaneous bridge module (NI Corporation, Laval, QC, Canada) via application of a bridge voltage of 2.5 V, then recorded with a sampling rate of 20 Hz. All measurements were repeated at least three times, and the output values were averaged before any of the graphs displayed in the main text were made. We confirmed that the measurement error was as low as 1% in most cases. The data-collection program was designed using LabVIEW (NI Corporation).

We inserted the strain gauge in the PDMS microchannel via cutting the PDMS edge right next to the inlet with a razor blade to make an opening; afterward, the opening was sealed tight with epoxy. A mass flow controller (MFC, SEC-N112MGM; Horiba Ltd., Kyoto, Japan) was used to regulate the flows of helium, nitrogen, air, argon, and carbon dioxide. Each flow was injected through the inlet at 50 mL/min for 10 s, followed by a 30 s interval without gas flow. The same gas-flow cycle was also performed at 75, 100, 125, and 150 mL/min, respectively. We used a volumetric flow meter (ProFLOW 6000 Electronic Flowmeter; Restek Corporation, Bellefonte, PA, USA) to measure the flow rates at the outlet and check for leakage.

## 4. Results and Discussion

The fabrication process of the device consisted of two stages: fabrication of a straight microchannel using PDMS and insertion of a strain gauge into the microchannel. The straight microchannel was fabricated through a typical micromolding procedure, and a photo of this microchannel is shown in Figure 1a. The height of the microchannel was observed to be 60 μm, as shown in Figure 1b. Then, a portion of the microchannel right next to the inlet was removed to make an opening for insertion of the strain gauge. The dimensions of the strain gauge are shown in Figure 1c. Since the thickest part of the strain gauge, where 130 μm-thick Cu electrodes were bonded on a 30 μm-thick epoxy film, was greater than the channel height, the top wall of the channel was deformed where the Cu electrodes were in contact with the PDMS, as schematically shown in Figure 1d. Considering that Young’s moduli of PDMS and epoxy are approximately 2 MPa and 3 GPa, respectively [32,33], while those of glass and Cu are roughly around 100 GPa each, we assume that only the PDMS and the epoxy were deformed by the insertion, but the deformation of the epoxy was much less than that of the PDMS. The device fabrication was finalized with a tight seal around the inlet, the outlet, and the opening, using epoxy, as shown in Figure 1e,f.

**Figure 2:**
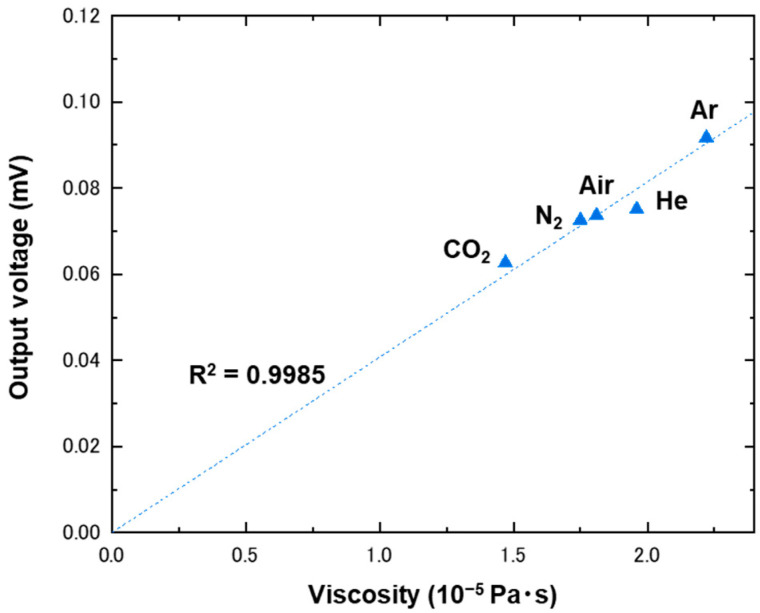
Plot of output voltages as a function of viscosity. The output voltages were measured using a strain gauge inserted in a microchannel with a height of 60 μm, under flows of CO_2_, N_2_, air, He, and Ar at 100 mL/min. The data points were fitted with a linear function, which has been drawn with a dashed line.

Unlike the correlation reported in previous studies [28,30,31,32], we show here, using the fabricated device, that there is almost a linear correlation between strain-gauge output voltages and the viscosities of five gases, including CO_2_, N_2_, air, He, and Ar, as shown in Figure 2. The output voltage was measured for each gas at a fixed flow rate of 100 mL/min. For quantitative interpretation, the R^2^ value was estimated via application of linear fitting. As a result, an R^2^ value of around 0.999 was obtained, meaning that gas viscosity can be determined via measurement of output voltage.

**Figure 3:**
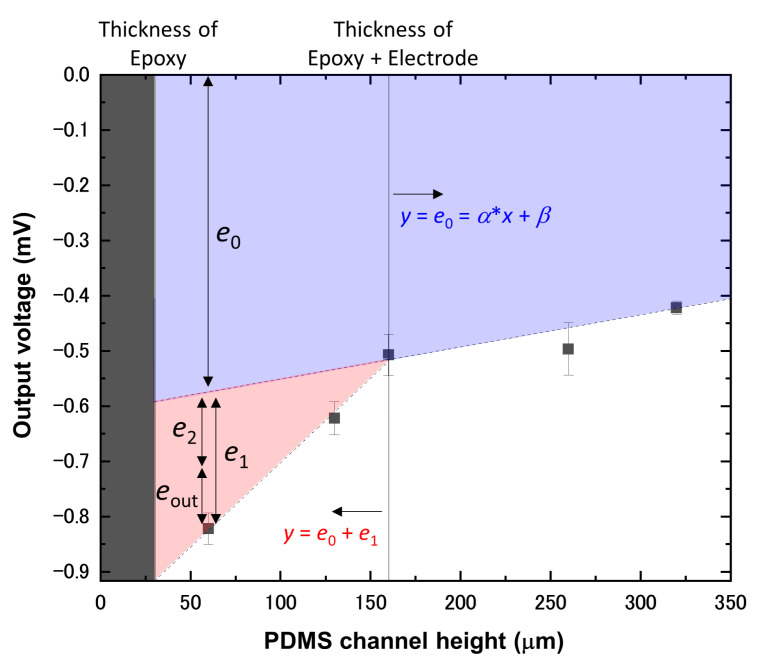
Plot of output voltages as a function of PDMS channel height. All of the output voltages were measured via insertion of a gauge into each microchannel without gas flow. The values for fitting parameters *a* and *b* were 5.81 × 10^−4^ and −0.609, respectively.

The output voltages under gas flow, $e_{out}$, were obtained as a result of the combination of several physical phenomena, induced via insertion of the strain gauge and the flowing gases: straightening of the strain gauge ($e_{0}$), strain generation induced via deformation of the channel wall ($e_{1}$), and flow-induced expansion of the channel ($e_{2}$). The insertion caused the first two. We kept carefully monitoring the change in output voltage when the strain gauge was inserted in the microchannel with 60 μm in height, without gas flow, since this height is much smaller than is the thickest part of the strain gauge. This mismatch made the strain gauge and the microchannel contact each other and was expected to generate some amount of output. In fact, we found that the insertion caused a negative voltage shift of 0.82 mV in the output, as shown in Figure 3. The strain gauge, which had a strain-detection film that was coated on its top surface, showed this negative shift when it experienced an upward deflection. As this deflection compressed the detection film, the negative shift corresponded to the compressive strain. Using microchannels with 130 and 160 μm in height, we observed that the shift in output voltage became smaller when the channel height became greater. This height dependence shows that the compressive strain decreased because the mismatch between the channel height and the thickness of the strain gauge also decreased. However, some shift still remained, even when the channel height reached 160 μm, which is equivalent to the thickest part of the strain gauge. The negative shift decreased further but fairly gradually from this height until the height increased up to 320 μm. Taking into account that the strain gauge was bending inherently—approximately 350–400 μm, as observed in Figure 1c—the gradual shift, in the range between the channel height of 160 and 320 μm, originated from the gauge straightening, namely $e_{0}$, caused by the insertion in a narrow channel. The amount of $e_{0}$ could be estimated for the microchannels that were shallower than 160 μm based on the linear fitting shown in Figure 3. We confirmed the validity of this approach via insertion of the strain gauge halfway into the microchannels and obtaining the linear relationship between the output voltages and the channel heights, as shown in Figure S2. The excess negative shift that was observed using microchannels with heights shallower than 160 μm should have been due to strain generation, namely $e_{1},$ induced via deformation of the channel wall. Unlike with $e_{0}$ and $e_{1}$, the measured $e_{out}$ values were all positive, as shown in Figure 2, suggesting that tensile strain is generated under gas flow. On the basis of these results, we hypothesize here that initially generated compressive strain via channel deformation ($e_{1}$) is partially released by channel expansion of under gas flow ($e_{2}$). In other words, $e_{out}$ is the difference between these two and is given with

(1) $$e_{out}=e_{1}-e_{2}$$

**Figure 4:**
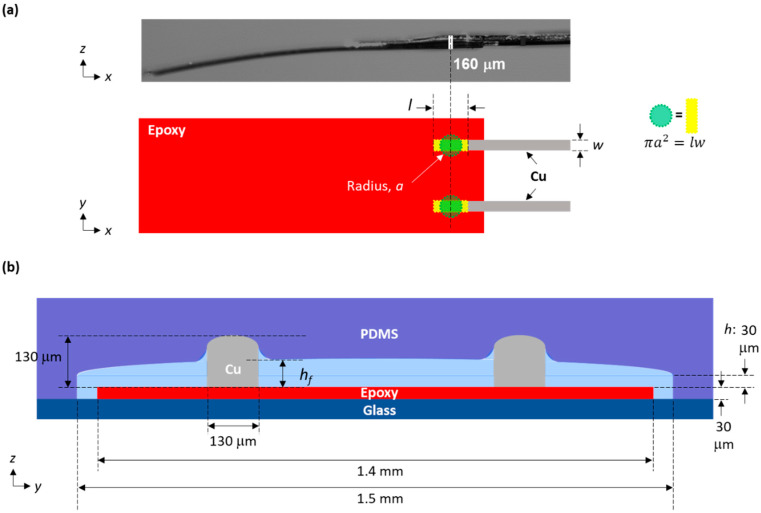
(**a**) Schematic of the area where indentation occurs on the strain gauge. This area has been drawn with two yellow rectangles whose length and width are l and w, respectively. We assumed that there were two cylinders whose circular cross-sections each had a radius of *a*, where the theory of indentation was applied. The cross-sectional area, $\pi a^{2}$, was set to be equivalent to that of the rectangle, lw. (**b**) Cross-sectional schematics of a microchannel with an inserted strain gauge under flow.

To elucidate the origin of these results, we discuss analytical details based on a schematic of the gauge inserted in the microchannel, shown in Figure 1d and Figure 4. All symbols and abbreviations used in the following discussion are listed in Table 1. When the strain gauge was inserted, the two 130 μm-thick Cu electrodes, bonded on a 30 μm-thick epoxy film, deformed the top surface of the 60 μm-thick PDMS channel, as depicted in Figure 1d. This deformation was almost entirely in the PDMS because the Young’s modulus of PDMS is significantly lower than that of Cu, glass, or the epoxy [34,35]. When a load, $F_{1}$, was applied to the PDMS due to an insertion of a depth of 130 [μm]-h, the theory of indentation using a cylindrical punch yields the following: [36]

(2) $$F_{1}=\frac{2aE}{\left(1-\nu^{2}\right)}\cdot \left(130-h\right)$$

where a is the cross-sectional radius of a circular punch, E*E* is the Young’s modulus of PDMS, ν is the Poisson’s ratio of PDMS, and h is the channel height after insertion of the epoxy film. Here, the Cu electrode is not a cylinder but a rectangle with a cross-sectional length of l and a width of w, so a must be modified. Assuming that the cross-sectional area for each shape is equivalent, as drawn in Figure 4a, a is given with

(3) $$a=\sqrt{\frac{lw}{\pi }}$$

Then, Equation (2) becomes

(4) $$F_{1}=\frac{2E\sqrt{lw}}{\sqrt{\pi }\left(1-\nu^{2}\right)}\cdot \left(130-h\right)$$

To estimate how much output voltage $\left(e_{1}\right)$ is generated when the strain gauge is inserted into the microchannel, we needed to calculate the dependence of $e_{1}$ on $F_{1}$. Since $F_{1}$ is applied on the epoxy film through the Cu electrodes, the film should be slightly deformed to have a strain, $\varepsilon^{\prime }$, normal to its surface. The resultant stress, σ, is obtained as follows:

(5) $$\sigma =\varepsilon^{\prime }E^{\prime }$$

where $E^{\prime }$ is the Young’s modulus of the epoxy [33]. Since stress is force per unit area, it can also be expressed as

(6) $$\sigma =\frac{F_{1}}{l\cdot w}$$

The strain gauge measures the strain, ε, in the lateral direction, given with

(7) $$\varepsilon =\nu^{\prime }\varepsilon^{\prime }$$

where $\nu^{\prime }$ is the Poisson’s ratio of the epoxy [37]. The output voltage of the strain gauge is obtained with [38]

(8) $$e_{1}=\frac{K_{s}e_{B}}{4}\cdot \varepsilon$$

where $K_{s}$ is the gauge factor and $e_{B}$ is the bridge voltage, both of which are known. Through combination of Equations (5)–(8), the relationship between $F_{1}$ and $e_{1}$ is given as

(9) $$F_{1}=\frac{4E^{\prime }lw}{K_{s}e_{B}\nu^{\prime }}\cdot e_{1}$$

Here, we combined Equations (4) and (9) to obtain

(10) $$e_{1}=\frac{K_{s}e_{B}\nu^{\prime }}{4E^{\prime }lw}\cdot F_{1}=\frac{EK_{s}e_{B}\nu^{\prime }\sqrt{lw}}{2\sqrt{\pi }E^{\prime }lw\left(1-\nu^{2}\right)}\cdot \left(130-h\right)=K\cdot \left(130-h\right)$$

For our microfluidic device, we used E = 1.75 MPa, which is measured for PDMS prepared under similar conditions: with the same weight ratio of base and curing agent (10:1) and heated at 65 °C for several hours [39]. The Poisson’s ratio for PDMS was taken to be ν = 0.499 [39]. Then, Equation (10) gave $e_{1}$ = 0.21 mV, which is in excellent agreement with the measured value of approximately 0.25 mV, as shown in Figure 3.

Then, we considered output voltage under flow $\left(e_{2}\right)$. When flow was introduced, the PDMS channel expanded due to the pressure induced via the flow. In the absence of electrodes, the channel would expand with a curved opening with a radius. Since we were only interested in the force applied to the electrodes, we assumed that the channel height at each electrode increased from h to $h_{f}$, as shown in Figure 4b. The load under flow, $F_{2}$, is given with

(11) $$F_{2}=\frac{2E\sqrt{lw}}{\sqrt{\pi }\left(1-\nu^{2}\right)}\cdot \left(130-h_{f}\right)$$

Thus, $e_{2}$ is given with

(12) $$e_{2}=\frac{K_{s}e_{B}\nu^{\prime }}{4E^{\prime }lw}\cdot F_{2}=K\cdot \left(130-h_{f}\right)$$

Now, through combination of Equations (1), (10), and (12), $e_{out}$ is given with

(13) $$e_{out}=\frac{K_{s}e_{B}\nu^{\prime }}{4E^{\prime }lw}\cdot \left(F_{1}-F_{2}\right)=K\cdot \left(h_{f}-h\right)$$

**Figure 5:**
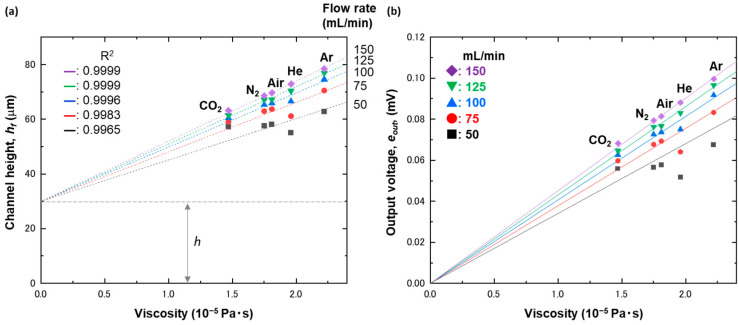
(**a**) Plot of channel heights under flow, $h_{f}$, as a function of viscosity. The data points were fitted with a linear function, which has been drawn with a dashed line. (**b**) Plot of output voltages, $e_{out}$, as a function of viscosity. The output voltages were measured using a strain gauge placed in a microchannel with a height of 60 μm, under flows of CO_2_, N_2_, air, He, and Ar at 50, 75, 100, 125, and 150 mL/min. The analytic model for each flow rate is shown with the solid lines.

Although $h_{f}$ is unknown, it should be dependent on viscosity, μ, and flow rate, Q [32]. Thus, we calculated $h_{f}$ values using the measured $e_{out}$ and examined the correlations with μ and Q. Similar to the trend shown in Figure 2, there was also a linear correlation between $h_{f}$ and μ, as shown in Figure 5a. Then, we found that the linearity became better as the flow rate increased up to 150 mL/min, whereas it became somewhat worse as the flow rate decreased to 50 mL/min. On the basis of the linear correlation, here, we describe $h_{f}$ as

(14) $$h_{f}\approx f\left(Q\right)\cdot \mu +h$$

where f(Q) is a flow-rate-dependent function. Through coupling of Equations (13) and (14), the relationship among $e_{out}$, f(Q), and μ is

(15) $$e_{out}=K\cdot f\left(Q\right)\cdot \mu$$

What this equation tells us is that $e_{out}$ is proportional to μ for a given f(Q). The linearity suggested by Equation (15) was experimentally confirmed, as shown in Figure 5b. The slope at each flow rate was estimated via linear fitting: 4.068, 4.304, and 4.515 at 100, 125, and 150 mL/min, respectively. The corresponding f(Q) values were then available through simple division of the slope by the constant, K. Finally, f(Q) is described as

(16) $$f\left(Q\right)=60.1\cdot Q^{0.257}$$

Therefore, the analytic model that correlates $e_{out}$ with Q and μ is given with

(17) $$e_{out}=60.1\cdot K\cdot Q^{0.257}\cdot \mu$$

As $e_{out}$ is proportional to the change in force with the flow of a fixed flow rate and thus the change in pressure, Δp, Equation (17) leads to

(18) Δp~C·μ

where C is the constant. Here, the linear correlation between the change in pressure and gas viscosity is provided. Unlike the reported correlations [28,30,32], our model is more viscosity-dependent, and thus, our approach achieved sensitivity high enough to even differentiate nitrogen and air, for which the difference in viscosities was 0.05 × 10^−5^ Pa·s. Moreover, we found that the present approach was sufficiently more accurate in estimation of gas viscosities than were the previous studies, as shown in Figure S3. Considering that the noise level of our measurements was less than 1 μV, we were able to differentiate structural isomers such as *n*-butane and isobutane, for which the difference in viscosities was 0.01 × 10^−5^ Pa·s, after optimizing several experimental conditions and carefully controlling critical parameters such as temperature and flow rate.

**Figure 6:**
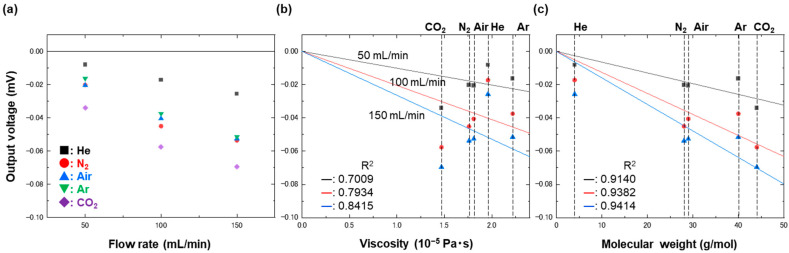
Plots of output voltages as a function of (**a**) flow rate, (**b**) viscosity, and (**c**) molecular weight. The output voltages were measured using a strain gauge placed in a microchannel with a height of 160 μm, under flows of CO_2_, N_2_, air, He, and Ar at 50, 100, and 150 mL/min. The data taken at different flow rates, shown in (**b**,**c**), were fitted with a linear function, which has been drawn with solid lines.

**Figure 7:**
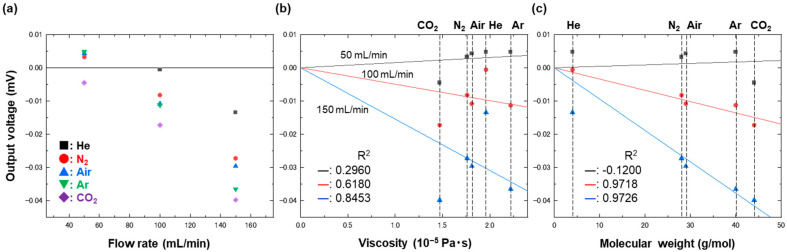
Plots of output voltages as a function of (**a**) flow rate, (**b**) viscosity, and (**c**) molecular weight. The output voltages were measured using a strain gauge placed in a microchannel with a height of 130 μm, under flows of CO_2_, N_2_, air, He, and Ar at 50, 100, and 150 mL/min. The data taken at different flow rates, shown in (**b**,**c**), were fitted with a linear function, which has been drawn with solid lines.

A possible reason why the experimental data show some deviation from the analytic model at low flow rate is due to a contribution other than viscosity. When a microchannel with a height of 160 μm—again, equivalent to the thickest part of the strain gauge—was used to measure the gases in the same manner, the results were completely different in terms of two facts, as shown in Figure 6; the output voltage was no longer viscosity-dependent but seemed to be rather molecular-weight-dependent, as shown in Figure 6b,c, and it showed negative values. The effect of molecular weight was reported previously using a microcantilever placed perpendicularly to a flow [40]. The same trend still held when the channel height of 130 μm was used, as shown in Figure 7. Since the epoxy film bent as observed in Figure 1c and failed to become perfectly flat after being inserted in the narrow channels, the flow could still have collided with the bending part to induce output voltage that could have been molecular-weight-dependent. The contribution of the molecular weight became smaller as the channel height became lower because the bending part became straighter, although it was still able to affect the output voltage when the channel height was 60 μm: double the film thickness.

The reason we observed negative values is that the channel height was comparable to the thickness of the strain gauge (160 μm), and thus, there was no initial compression due to contact between the PDMS and the Cu. What occurred when gas was injected was flow-induced compression of the strain gauge, as shown in Figure S4. Due to this compression, the resultant output voltage was negative, as shown in Figure 6. This explanation is validated by the result shown in Figure 7. These data were obtained using a microchannel of 130 μm in height, which is slightly smaller than the thickness of the strain gauge. With this configuration, we obtained positive output values when the flow rate was as low as 50 mL/min; however, we then obtained negative output values when the flow rate increased. These results indicate that pressure release occurred at low flow rates, but once the channel expanded more and there was no longer any contact between the PDMS and the Cu at higher flow rates, the output voltage became negative. To ensure long-term stability of the device within the viscosity-dependent regime, the device needs to have a channel height much greater than the thickness of the strain gauge and to be used at a moderate flow rate that will not induce too much deformation, without significant measurement-to-measurement variations.

**Figure 8:**
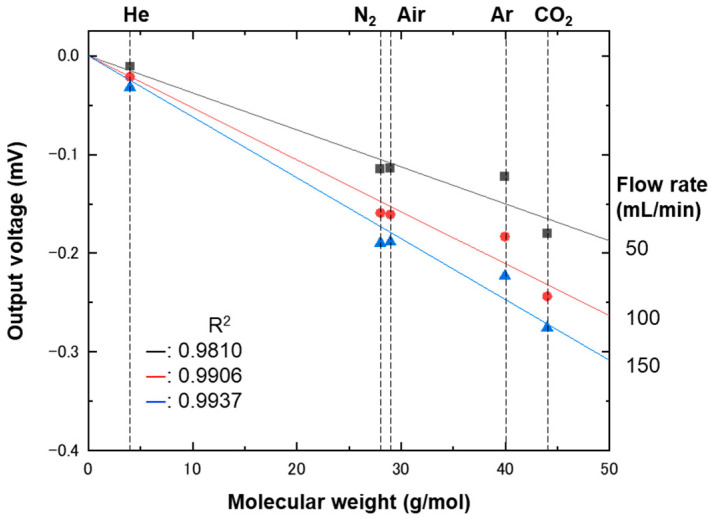
Plot of output voltages as a function of molecular weight. The output voltages were measured using a strain gauge placed perpendicularly to a flow in a microchannel with a height of 160 μm. Five gases—helium, nitrogen, air, argon, and carbon dioxide—flowed at 50 (squares), 100 (circles), and 150 (triangles) mL/min. The data taken at different flow rates were fitted with a linear function, which has been drawn with solid lines.

According to the previous study [40], the contribution of molecular weight should be separated from that of viscosity via putting the strain gauge not parallel but perpendicular to the flow. To confirm, the strain gauge was placed perpendicularly to the flow in the microchannel with a height of 160 μm. As a result, clear molecular-weight dependence was observed, as shown in Figure 8. It should be emphasized here that simply changing gauge configuration from a parallel to a perpendicular flow provides totally different information, i.e., viscosity and molecular weight. Therefore, this approach could even be used to discriminate structural isomers, the discrimination of which usually requires mass spectrometry.

## 5. Conclusions

The viscosities of gases are found to linearly correlate with strain-gauge output voltages that are transduced into pressure. A gauge inserted in a 60 μm-high PDMS microchannel deformed the top surface of the channel because of a 130 μm-thick part of the gauge. This deformation caused some initial pressure on the gauge. The introduction of flow expanded the channel, resulting in a decrease in initial pressure. The difference in pressure between the two conditions—without and under flow—linearly correlated with gas viscosity in the range from 1.47 to 2.22 × 10^−5^ Pa·s. With the increasing demand for rapid, facile measurements using mobile devices, even outside laboratories, this technique would enable us to measure any gas and thus to discriminate gases based on their viscosities, leading to various applications in safety, food, cosmetics, health, and related fields.

## Figures and Tables

**Table 1:** List of symbols/abbreviations.

Symbol or Abbreviation	Detail
$F_{1}$	Load applied to PDMS due to insertion
a	Cross-sectional radius of a circular punch
E	Young’s modulus of PDMS
ν	Poisson’s ratio of PDMS
h	Channel height after insertion of the epoxy film
l	Cross-sectional length of a rectangle
w	Cross-sectional width of a rectangle
$\varepsilon^{\prime }$	Strain normal to the epoxy surface
σ	Stress applied to the epoxy surface
$E^{\prime }$	Young’s modulus of the epoxy
ε	Strain in the lateral direction
$\nu^{\prime }$	Poisson’s ratio of the epoxy
$K_{s}$	Gauge factor
$e_{B}$	Bridge voltage
$h_{f}$	Channel height under gas flow
$F_{2}$	Load under gas flow
μ	Viscosity of a gas
Q	Flow rate
f(Q)	Flow-rate-dependent function
Δp	Induced pressure change via gas flow

## Data Availability

The data presented in this study are available on request from the corresponding authors.

## Supplementary Materials

The following supporting information can be downloaded at: https://www.mdpi.com/article/10.3390/bios13010076/s1, Figure S1: Schematic of a bridge circuit used in this study; Figure S2: Plot of output voltages as a function of PDMS channel height; Figure S3: Predicted viscosity as a function of known viscosity for various gases; Figure S4: Schematic of the strain gauge inserted in a microchannel with a height of 160 μm.
