# Supplementary material for: Strain Sensor-Inserted Microchannel for Gas Viscosity Measurement
Source: Biosensors (Basel). 2023 Jan 1;13(1):76. doi: 10.3390/bios13010076 (PMC9855327; doi:10.3390/bios13010076)
Supplement: Supplementary file 1 [file biosensors-13-00076-s001.zip › biosensors-2000090-supplementary.pdf]

Strain Sensor-Inserted Microchannel for Gas Viscosity Measurement

Kota Shiba [1,2,*], Linbo Liu [2,3] and Guangming Li [2,4]

[1] Center for Functional Sensor & Actuator (CFSN), National Institute for Materials Science (NIMS), 1-1 Namiki, Tsukuba, Ibaraki 305-0044, Japan
[2] John A. Paulson School of Engineering and Applied Sciences, Harvard University, 9 Oxford Street, Cambridge, MA 02138, USA
[3] DAAN Gene Co., Ltd. of Guangzhou, 19 Xiangshan Road, Science Park, High & New Technology Development District, Guangzhou 510665, China
[4] State Key Laboratory of Rare Earth Resource Utilization, Changchun Institute of Applied Chemistry, 5625 Renmin Street, Changchun 130022, China
* Correspondence: SHIBA.Kota@nims.go.jp; Tel.: +81-29-860-4603

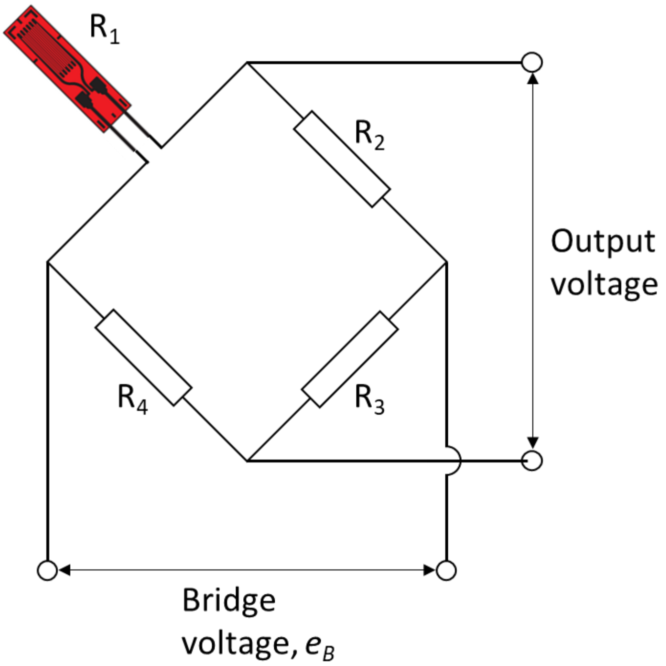

Figure S1. Schematic of a bridge circuit used in this study.

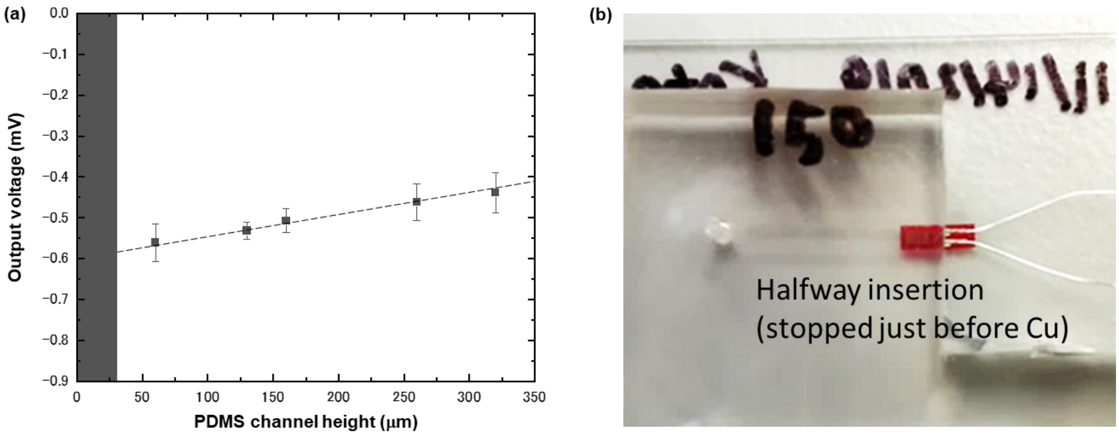

Figure S2. (a) Plot of output voltages as a function of PDMS channel height. All the output voltages are measured by inserting the strain gauge halfway in a microchannel with a height of 60, 130, 160, 260, and 320 μm. The data taken at different flow rates are fitted with a linear function which is drawn with the dashed lines. (b) Photo of the strain gauge inserted halfway in the microchannel.

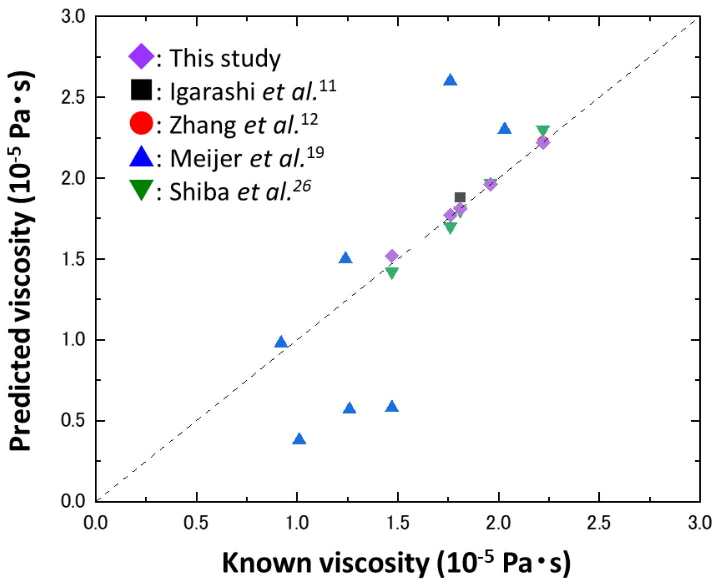

Figure S3. Predicted viscosity as a function of known viscosity for various gases. The data shown in this paper is compared to those reported previously.

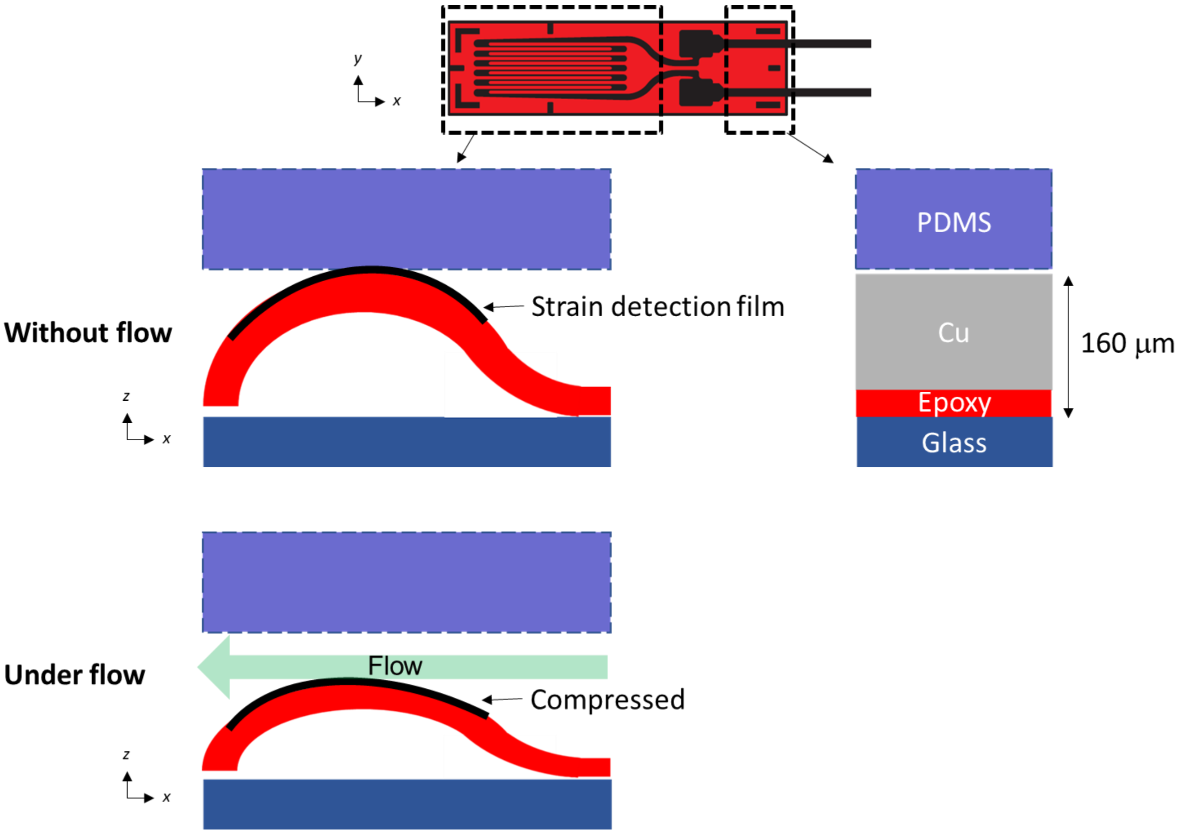

Figure S4. Schematic of the strain gauge inserted in a microchannel with a height of 160 μm (top) without flow and (bottom) under flow.
